# Cavitation and water fluxes driven by ice water potential in *Juglans regia* during freeze–thaw cycles

**DOI:** 10.1093/jxb/erv486

**Published:** 2015-11-19

**Authors:** Katline Charra-Vaskou, Eric Badel, Guillaume Charrier, Alexandre Ponomarenko, Marc Bonhomme, Loïc Foucat, Stefan Mayr, Thierry Améglio

**Affiliations:** ^a^INRA,UMR PIAF, F-63100 Clermont-Ferrand, France; ^b^Clermont Université, Blaise Pascal University, UMR PIAF, F-63100 Clermont-Ferrand, France; ^c^Department of Botany, University of Innsbruck, A-6020 Innsbruck, Austria; ^d^INRA UR BIA, F-44316 Nantes, France

**Keywords:** Acoustic emissions, bark shrinkage, embolism, *Juglans regia* L., microdendrometer, walnut tree, winter biology, X-ray microtomography.

## Abstract

Freeze–thaw cycles induce major hydraulic changes due to liquid-to-ice transition within tree stems. The very low water potential at the ice–liquid interface is crucial as it may cause lysis of living cells as well as water fluxes and embolism in sap conduits, which impacts whole tree–water relations. We investigated water fluxes induced by ice formation during freeze–thaw cycles in *Juglans regia* L. stems using four non-invasive and complementary approaches: a microdendrometer, magnetic resonance imaging, X-ray microtomography, and ultrasonic acoustic emissions analysis. When the temperature dropped, ice nucleation occurred, probably in the cambium or pith areas, inducing high water potential gradients within the stem. The water was therefore redistributed within the stem toward the ice front. We could thus observe dehydration of the bark’s living cells leading to drastic shrinkage of this tissue, as well as high tension within wood conduits reaching the cavitation threshold in sap vessels. Ultrasonic emissions, which were strictly emitted only during freezing, indicated cavitation events (i.e. bubble formation) following ice formation in the xylem sap. However, embolism formation (i.e. bubble expansion) in stems was observed only on thawing via X-ray microtomography for the first time on the same sample. Ultrasonic emissions were detected during freezing and were not directly related to embolism formation. These results provide new insights into the complex process and dynamics of water movements and ice formation during freeze–thaw cycles in tree stems.

## Highlight

The dynamics of water fluxes and cavitation events induced by ice formation were analyzed by visualization (X-ray microtomography and MRI), and detected with a microdendrometer and ultrasonic acoustic emissions.

## Introduction

Freezing events are a limiting factor for plant survival and distribution in temperate, alpine, and boreal biomes (Sakai and Larcher, 1987; Charrier *et al.*, 2013a). Injuries generated by freezing temperatures can cause the death of plants (Burke *et al.*, 1976; Sakai and Larcher, 1987; Cinotti, 1989; Charrier *et al.*, 2015a) or impair their reproductive success (Rodrigo, 2000; Chuine and Beaubien, 2001). In trees, typically two types of damage can be generated by freeze–thaw stress, which affect the hydraulic integrity (Charrier *et al.*, 2013a) and survival (Sakai and Larcher, 1987; Neuner and Pramsohler, 2006; Hacker and Neuner, 2008) of the plant: vessel embolism (Utsumi *et al.*, 1999; Feild and Brodribb, 2001; Cruiziat *et al.*, 2002; Ball *et al.*, 2006) and lysis of living cells (Mazur, 1984; Charrier *et al.*, 2013b).

Ice nucleation creates a high water driving force toward the ice front, inducing major water fluxes within the stem (Palta *et al.*, 1977; Cinotti, 1989, 1991; Steponkus and Webb, 1992) or leaf (Ball *et al.*, 2006). The very low water potential (Ψ) of ice at any given freezing temperature can be calculated from the Clausius–Clapeyron relationship (Rajashekar and Burke, 1982; Rajashekar *et al.*, 1983; Guy, 1990):

$$\Delta \Psi_{T}=–\text{1.16}T$$ (1)

where ΔΨ_*T*_ (MPa) is the difference in Ψ between two compartments at temperature *T*, and where *T* (° C) is the temperature below the effective freezing point. This equation shows that living cells are excessively dehydrated by decreasing temperatures (–1.16MPa K^−1^). Low Ψ induced by ice can therefore explain the significant shrinkage of the stem detected during freezing events (Cinotti, 1989; Zweifel and Häsler, 2000; Améglio *et al.*, 2001; Sevanto *et al.*, 2006). It may also be sufficient to induce cavitation and embolism within xylem conduits (Sucoff, 1969; Ewers, 1985; Robson *et al.*, 1988).

Intracellular ice formation leads to the death of living cells in the stem (Mazur, 1963, 1969; Guy, 1990; Andrews, 1996; Wolfe and Bryant, 2001). At low freezing rates or in acclimated stems, ice nucleation occurs in the apoplastic compartment. Extracellular ice causes a drastic dehydration and induces osmotic stress affecting the cytoplasm and membranes of living cells (Gusta *et al.*, 1975; Steponkus, 1984; Ruelland *et al.*, 2009), but, due to extracellular freezing, not necessarily the death of cells.

Xylem water transport capacity is impacted by freeze–thaw cycles when winter embolism is induced in xylem conduits (Sperry and Sullivan, 1992; Tyree *et al.*, 1994; Hacke *et al.*, 2001; Mayr *et al.*, 2007), but the mechanisms and dynamics of this phenomenon are still unclear. The ‘thaw–expansion hypothesis’ (Ewers, 1985; LoGullo and Salleo, 1993; Lemoine *et al.*, 1999; Hacke and Sperry, 2001; Tyree and Zimmermann, 2002; Pittermann and Sperry, 2003, 2006) postulates that gas bubbles are formed in conduits when sap freezes, because air is barely soluble in ice. During thawing, these bubbles expand when the pressure of the surrounding sap becomes sufficiently negative to counter the bubble-collapsing force of surface tension (Pittermann and Sperry, 2006). Several experimental studies are consistent with this ‘thaw–expansion hypothesis’ with observations of bubbles during freezing (Sucoff, 1969; Ewers, 1985; Robson *et al.*, 1988), embolism formation linked to vessel diameter (Davis *et al.*, 1999; Sperry and Robson, 2001; Pittermann and Sperry, 2003; Pittermann and Sperry, 2006; Charrier *et al.*, 2013a, 2014a), or negative xylem sap pressure as a precondition to induce embolism formation (Sperry and Sullivan, 1992; Langan *et al.*, 1997; Davis *et al.*, 1999; Sperry and Robson, 2001; Améglio *et al.*, 2002; Mayr *et al.*, 2003; Pittermann and Sperry, 2006; Stuart *et al.*, 2007; Mayr and Sperry, 2010). Nevertheless, other studies have contradicted the ‘thaw–expansion hypothesis.’ First, several studies have shown that the rate of embolism increased with the number of freeze–thaw cycles (Sperry *et al.*, 1994; Sparks and Black, 2000; Sparks *et al.*, 2001; Mayr *et al.*, 2003, 2007; Mayr and Zublasing, 2010; Charra-Vaskou *et al.*, 2012b), although all conduits of critical size should cavitate in the first freezing cycle. Secondly, a lack of correlation between conduit size and intrinsic vulnerability was found (Mayr *et al.*, 2007). According to the ‘thaw-expansion hypothesis,’ the probability of cavitation increases with conduit size as larger bubbles are formed during freezing. Thirdly, ultrasonic emissions (UEs), commonly related to embolism, were registered during freezing (Kikuta and Richter, 2003; Mayr *et al.*, 2007; Mayr and Sperry, 2010), while embolism should occur during thawing.

Measurement of UEs is a standard method for drought-induced embolism monitoring in the laboratory (Salleo and Lo Gullo, 1986; Borghetti *et al.*, 1993; Salleo *et al.*, 2000; Mayr and Rosner, 2011; Vergeynst *et al.*, 2014) and in the field (Jackson and Grace, 1996; Höllta *et al.*, 2005; Ogaya and Penuelas, 2007). During freeze–thaw cycles, UEs are emitted on freezing (Weiser and Wallner, 1988; Raschi *et al.*, 1989; Kikuta and Richter, 2003; Mayr *et al.*, 2007; Mayr and Sperry, 2010; Mayr and Zublasing, 2010; Kasuga *et al.*, 2015). Charrier *et al.* (2014a) observed a significant correlation between UEs and loss of hydraulic conductivity after a freeze–thaw cycle. The authors hypothesized that UEs were not correlated with embolism but rather were emitted by bubble formation in the freezing sap (‘freeze cavitation hypothesis’; see also Charrier *et al.*, 2015b), as observed previously within artificial vessel devices (Ponomarenko *et al.*, 2014). In contrast, bubble expansion within conduits (embolism formation) was suggested to occur on thawing. Embolism was observed previously during thawing by cryoscanning electron microscopy in *Betula platyphylla* and *Salix sachalinensis* (Utsumi *et al.*, 1998) and *Fraxinus mandshurica* (Utsumi *et al.*, 1999) and by Ball *et al.* (2006) on leaves. However, to date, embolism formation during freeze–thaw cycle has never observed on the same sample. In this study, a direct measure of embolism formation via X-ray microtomography during freeze–thaw cycles on the same sample allowed us to test both hypotheses and to analyze the dynamics of embolism formation.

The main objective of this study was to understand and to visualize the hydraulic processes during successive events of freezing and thawing. We hypothesized that ice nucleation is located in the cambium and/or pith areas (Améglio *et al.*, 2001; Ball *et al.*, 2006) and that it generates spatial heterogeneity in Ψ. Water would be attracted toward the site of ice nucleation, leading to dehydration of the bark and xylem, so that cavitation thresholds are reached in xylem conduits (‘freeze cavitation hypothesis’). On thawing, we expected embolism formation, according to the ‘thaw–expansion hypothesis’, to concur with the two current hypotheses discussed. We combined four complementary tools: (i) microdendrometers to monitor the stem diameter and indicate the radial water fluxes during freeze–thaw events (Zweifel *et al.*, 2000; Améglio *et al.*, 2001, 2003); (ii) nuclear magnetic resonance imaging (MRI) allowing visualization of the liquid water allocation before and after freeze–thaw cycles (Faust *et al.*, 1997; Holbrook *et al.*, 2001; Clearwater and Clark, 2003; Van As, 2007); (iiii) X-ray microtomography to visualize embolism inside plants, and also during freezing; this has been performed previously with drought-stressed plants (Brodersen *et al.*, 2010; Charra-Vaskou *et al.*, 2012a; Dalla-Salda *et al.*, 2014; Torres-Ruiz *et al.*, 2014), and is now becoming a reference technology in order to measure embolism in plants without cutting artifacts (Wheeler *et al.*, 2013; Cochard *et al.*, 2014); and (iv) UE measurement to analyze the dynamics of cavitation events (Ponomarenko *et al.*, 2014) during freeze–thaw cycles (Charrier *et al.*, 2015b).

## Materials and methods

### Plant material

Plant material was sampled in the INRA PIAF orchard, site de Crouël, Clermont-Ferrand, France (350 m above sea level, 45°46′ N, 3°04′ E). Samples from current and 1-year-old branches were collected from sun-exposed branches of walnut trees (*Juglans regia* L. cv. Franquette) in winter. Branches of about 40cm in length were cut, immediately wrapped in plastic bags, transferred to the laboratory, rehydrated overnight, and cut again under water the day after (about 30cm long) in order to gradually release the xylem tension. Branches were shortened under water to obtain 14 final samples of around 10cm in length and 1cm in diameter for all experiments, and three samples of around 25cm in length for MRI measurements. Both ends were rapidly soaked in liquid paraffin wax to seal vessel ends, and samples were wrapped in Parafilm (Pechiney Plastic Packaging, Chicago, IL, USA) to avoid dehydration.

### Temperature treatments (except for MRI imaging)

Temperature treatments were performed within a temperature-controlled chamber (Binder GmbH, Tuttlingen, Germany) over 37h. The temperature protocol contained one freeze–thaw cycle (+5 °C, down to –40 °C, up to +8 °C) with a rate of 5K h^−1^ over 1h, followed by a 1h step every 5 °C (Table 1). This minimum temperature of –40 °C allowed us to stop and avoid all active mechanism and any physiological reactions (such as possible refilling) in order to focus on physical phenomena (plasmolysis or embolism) induced by the low ice water potential. This temperature dynamics is a reference treatment to study the effects of freeze–thaw cycles (Charrier *et al.*, 2014a; Kasuga *et al.*, 2015). The final temperature of +8 °C was also chosen to avoid active refilling and stem pressure in samples (Sperry *et al.*, 1987; Améglio *et al.*, 1995; Hacke and Sauter, 1996; Holbrook and Zwieniecki, 1999; Améglio *et al.*, 2001). Control samples were kept at +5 °C for 37h in chambers computer controlled by a circulator bath (Ministat Huber, Offenburg, Germany: –25 °C to +120 °C). The temperature in chambers was controlled using thermocouples and recorded every 1s with a data logger (DL2e, Delta T devices, UK).

**Table 1.** Characteristics, indications, and observations provided by the different techniques used in the study The type of measurement (dynamic or static), type of method (direct or indirect), indications given by each technique, temperature measurements (frequency in the case of dynamic measurement and temperature of measurement in the case of static measurements), minimum temperature reached during the temperature treatment, observations/interpretations with each technique in this study, and related figures for the four techniques used: microdendrometer, MRI, X-ray microtomography, and UEs, are given.

Techniques used	Type of measurement	Type of method	Observation	Temperature measurements	Temperature cycles	Interpretations	Figure(s)
Microdendrometer	Dynamic	Indirect observation	• Damage to living cells • Radial shrinkage/ swelling linked to water fluxes	Continuous monitoring	• +5 to –40 °C (–5 °K.h^−1^) • 1h at –40 °C • –40 to +8 °C (5 °K.h^−1^)	• Shrinkage of the stem indicating dehydration of living cells and radial water fluxes between around –5 and –15 °C • Radial water fluxes toward the bark during thawing from around –15 to 0 °C	1
MRI	Static (before and after freeze–thaw cycle)	Direct visualization	• Free water spatial distribution • Embolized vessels	+15 °C	• +5 to –10 °C (–12.5K h^−1^) • 1h at –10 °C • –10 to +15 °C (12K h^−1^)	• Higher free water content in cambium zone coming from the wood • Embolized vessels • No difference in free water content in bark	3, 4, 8
X-ray microtomography	Static (before and after freeze–thaw cycle, as well as in frozen state)	direct visualization	• Tissue dimensions • Internal structure • Embolized vessels	+5 °C –40 °C +8 °C	• +5 to –40 °C (–5K h^−1^) • 1h at –40 °C • –40 to +8 °C (5K h^−1^)	• Stem shrinkage during freezing mainly located in bark • Embolism formation during thawing	2, 5, 6
UE	Dynamic	Indirect observation	• Cavitation events	Continuous monitoring	• +5 to –40 °C (–5K h^−1^) • 1h at –40 °C • –40 to +8 °C (5K h^−1^)	• Cavitation events during freezing from around *–*5 to *–2*5 °C	7

### Microdendrometer

**Fig. 1.**
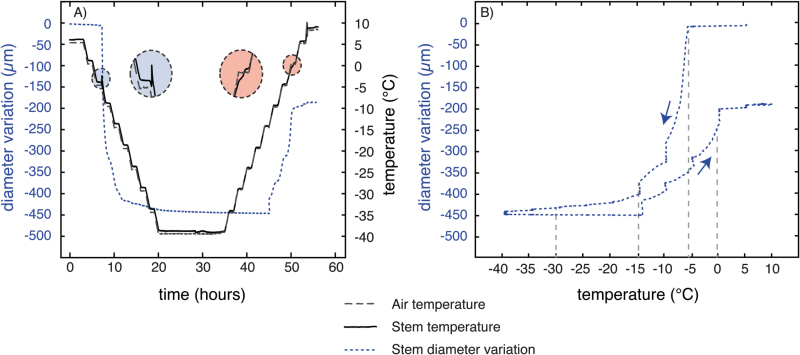
Stem diameter variation during one freeze–thaw cycle. Diameter variation was recorded on three stem samples with a PépiPIAF over one freeze–thaw cycle (+5 °C/–40 °C/+8 °C). The diameter variation of a single sample is shown and is representative of the three samples. Data acquisition was every 1min (sensitivity±1µm, PépiPIAF system, Forest Future, Nancy, France).

Stem diameter variations were monitored on three samples during one freeze–thaw cycle down to –40 °C, as described above. The diameter variation of a single sample is shown in Fig. 1 and is representative of the three samples. We used a special linear variable differential transformer sensor with data acquisition every 1min (sensitivity±1 µm; PépiPIAF system, Forest Future, Nancy, France).

### MRI imaging

The MRI observations were performed at INRA, Clermont-Ferrand, France, on an Avance DRX-400 micro-imaging system (Bruker, GmbH, Ettlingen, Germany) with a wide-bore (89mm) vertical 9.4 T magnet and an actively shielded gradient coil allowing a maximum gradient strength of 1000 mT m^−1^. The samples were placed in a 30mm diameter birdcage radiofrequency coil used for both excitation and signal reception. During MRI measurements, the temperature was monitored (±0.5 °C) inside the probe. For freeze–thaw treatment, a cooling and warming rate of 12.5K h^−1^ was applied and maintained at –10 °C for 1h (Table 1). All MRI images (before and after the freezing process) were recorded at stabilized temperature of 15 °C. Below 0 °C, as the water was in a solid state (ice), no scans were performed as a signal was not detectable.

Due to the rapid decline of the water MRI signal (characterized by a transverse relaxation time *T*_2_ of ≤1ms, estimated from the line width of the water peak of about 300 Hz at 15 °C), a gradient echo sequence with a minimum echo time of 2.6ms was used for image acquisition. To reduce the acquisition time and to optimize the signal-to-noise ratio, a short repetition time of 150ms and an excitation pulse at a 30° tip angle were used. Four contiguous transverse slices (perpendicular to the stem axis) were acquired simultaneously in sequential mode. Each slice was 3mm thick. Images were obtained with a 1.28×1.28cm^2^ field of view. The images were 256×256 pixels, giving an in-plane resolution of 50×50 µm^2^. The acquisition time was 30min. Images were acquired before (*t*=0) and after (*t*=6h) the freezing/thawing process on two treated samples and one control sample.

### Three-dimensional X-ray microtomography

The 10cm long samples were scanned with an X-ray microtomograph (Nanotom 180 XS, GE, Wunstorf, Germany) at the PIAF laboratory (INRA, Clermont-Ferrand, France). This method is based on the local X-ray absorption behavior of the sample mainly according to the local density. It provides direct observation of the internal structure of an intact sample without surface or cutting preparation (Brodersen *et al.*, 2010; Charra-Vaskou *et al.*, 2012a; Dalla-Salda *et al.*, 2014; Torres-Ruiz *et al.*, 2014). Eight samples were observed: five were submitted to one freeze–thaw cycle (see above). X-ray microtomographic scans were recorded before (+5 °C), after (+8 °C) and at the minimum point (–40 °C) of the freeze–thaw cycle (Table 1). The three other samples were kept at a constant temperature (+5 °C) during the whole experiment (control samples). They were also scanned at the same time as the treated samples (*t*=0, 18, and 36h). During the temperature course, samples were taken three times out of the temperature-controlled chamber, weighed, and immediately inserted within a polystyrene insulating cylinder for the X-ray scan. The sample temperature could not be measured during X-ray scans, but the quality of images showed that no changes in dimension occurred during acquisitions, suggesting that the sample stayed frozen during scans. Furthermore, no variation in sample weight was observed before and after scanning, indicating no sample water losses (data not shown).

The scanning setup was adjusted in order to manage the trade-off between the spatial resolution and the limitation of the sample heating during the scan. The field of view was adjusted in order to cover the whole sample cross-section. The spatial resolution was 11×11×11 µm^3^ per voxel. The X-ray parameters were 60kV and 200 µA. For each sample, 600 images were recorded during a 360° rotation of the sample. A fast scan time was fixed at 13min. Full three-dimensional volumes were reconstructed by datos|× 2.0 software (Phoenix, Nanotom 180 XS, GE, Wunstorf, Germany). Volumetric image analysis and visualization were performed using VGStudio Max^©^ 2.1 software (Volume Graphics, Heidelberg, Germany). After each scan, samples were removed from the insulating cylinder, weighed again, and put back into the temperature-controlled chamber. Samples were kept out of the chamber for less than 15min.

### Image analysis

MRI analysis was performed using MatLab (MathWorks, Massachusetts, USA). Since the contrast between the vessels filled with water and the other areas was very high, a threshold process was used to allow us to discriminate between water-filled and air-filled vessels. The threshold value was the same for all of the treated images. The area and the major and minor axes of an elliptic approximation were measured. The equivalent diameter was computed for every vessel based on its area ($D_{eq}=2\sqrt{\frac{A}{\pi }}$ , where *D*_*eq*_ is the equivalent diameter and *A* is the area of vessel cross-section). The number of water-filled vessels and the size distribution of the vessel diameters were measured. The grey level intensity, which refers to the amount of water, was measured for outer bark, inner bark, pitch, and the cambium–phloem zone to follow the evolution of the water signal intensity. The means and standard errors of intensity were calculated in each area based on individual pixels. X-ray microtomography scan analysis (*n*=5) was performed with ImageJ software to determine stem diameter and tissue thickness variations of the bark, wood, and pith, as well as the percentage of embolism before (+5 °C), during (–40 °C), and after (+8 °C) freeze–thaw cycles. For controls (*n*=3), tissue thickness and embolisms were determined on scans done at *t*=0, 18 and 36h, which corresponded to each temperature step.

### UEs

Three stem samples were subjected to one freeze–thaw cycle down to –40 °C (see above), while UEs (Mayr and Sperry, 2010; Mayr and Zublasing, 2010; Mayr and Rosner, 2011; Charrier *et al.*, 2014b, 2015b) were recorded (Table 1).

One ultrasonic sensor (150 kHZ resonance sensor, R15/C, 80–400kHz) was attached to each samples. In the middle of the sample (10cm in length), 3cm^2^ of the Parafilm and bark were removed and the xylem was covered with silicone grease in order to improve acoustic coupling and prevent evaporation. R15C sensors were then attached with clamps (plastic-coated metal springs). Sensors were connected to a 20/40/60 dB preamplifier set to 40 dB and plugged into a SAMOS device (SAMOS PAC 125 18-bit A/D, 3kHz to 3 MHz PCI2). All components of the UE system were supplied by Mistras (Sucy-en-Brie, France).

The acoustic detection threshold was set at 45 dB. Peak definition time, hit definition, and hit lockout times were 200, 400, and 2 µs, respectively. Recording analysis of UEs used AEwin software (Mistras).

For vulnerability analysis, the cumulative number of ultrasonic emissions during temperature treatments was related to the total number of UEs until all acoustic activity stopped.

### Statistics

All values are given as means±standard error (the number of samples is given in the text). Differences between sample sets were tested with Student’s *t*-test after testing for Gaussian distribution (Kolmogorov–Smirnov test) and variance homogeneity (Levene test) of data. In the case of heterogeneous variances, Welch’s test was used. All tests (two-tailed) were performed pairwise at a probability level of 5% using SPSS (version 15.0; SPSS Inc, Chicago, IL, USA).

## Results

### Diameter and tissues thickness variations

**Fig. 2.**
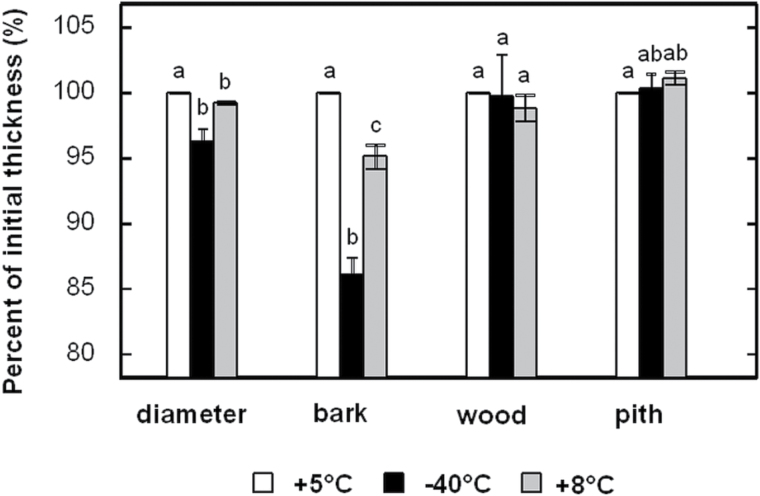
Thickness variations of stem tissues during the freeze–thaw cycle. The thickness of the bark, wood, and pith, as well as the diameter, were measured from X-ray microtomography pictures using image analysis software (ImageJ). Image acquisitions of stems (*n*=5) at +5 °C (before, *t*=0), during (–40 °C, *t*=18h), and after (+8 °C, *t*=36h) the freeze–thaw cycle were used. Results are shown as means±SE (*n*=5). Bars with a different letter differed significantly at *P*≤0.05.

During freezing, immediately after the exotherm was detected (–5 °C; Fig. 1), a drastic radial shrinkage of stem diameter was observed (*~*54 µm K^−1^ between –5 °C and –10 °C). A shrinkage of lower intensity was observed between –10 and –15 °C (*~*9 µm K^−1^). The stem diameter then tended to stabilize (~1.8 µm K^−1^ between –15 and –40 °C; Fig; 1) reaching 96% of the initial size at –40 °C (Fig. 2).

During thawing, no stem diameter variation occurred between –40 and –15 °C, but it suddenly increased at a temperature higher than –15 °C. Stem diameter variation was related to temperature steps but stopped after the endotherm was observed (*~*+6 °C; Fig. 1). Between –40 and +8 °C, stem diameter recovered to 99% of initial diameter (Figs 1 and 2).

X-ray microtomography observations provided new information at the tissue level: neither wood nor pith showed any significant thickness variation. Stem diameter variation during the freeze–thaw cycle was mostly due to changes in the bark. Indeed, a diameter shrinkage of about 3.7% was observed (96.3% of its initial thickness at –40 °C; Fig. 2) in the sample about 8200 µm in diameter, i.e. a shrinkage of 303 µm. A bark shrinkage of 13.9% was observed (86.1% of initial thickness at –40 °C; Fig. 2). The bark measured about 930 µm, which corresponded to a shrinkage of 129 µm (this had to be multiplied by 2 for both sides of the bark). During freezing, the bark shrinkage was then about 258 μm when the total diameter shrinkage was about 303 µm. The relative bark shrinkage (13.9%) thus explained almost the entire relative diameter variation of the stem (3.7%). We observed incomplete recovery on thawing with a loss in diameter of almost 200 µm (*~*2% of initial size) after the freeze–thaw cycle down to –40 °C (Fig. 1).

### MRI image observations

**Fig. 3.**
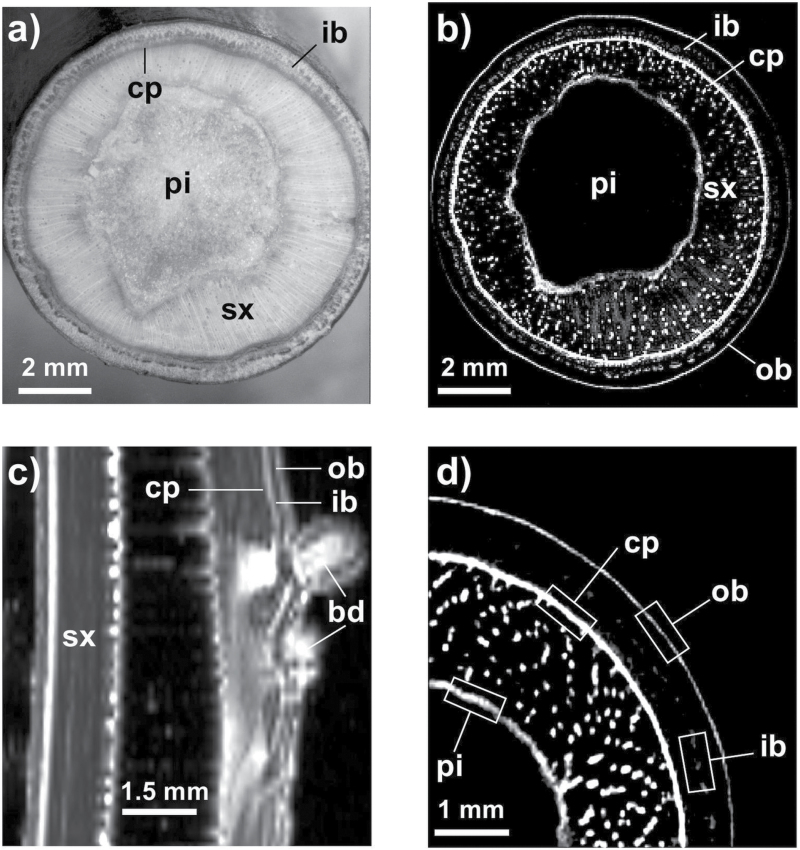
Light microscopy and MRI micrograph comparisons of transverse and longitudinal sections of 1-year-old stem of *J. regia* L. (a) Transverse light microscopy section. (b) Transverse MRI image of the same stem as in (a). (c) Longitudinal MRI image of a stem with an axial bud. (d) Detail from a transverse MRI image of the same stem as in (b). pi, Pith; sx, secondary xylem; ob, outer bark; ib, inner bark; cp, cambium–phloem zone; bd, bud. The four rectangles in (d) represented the four different zones used for MRI signal intensity analysis (related to free water content).

MRI images depicted free liquid water distribution within samples at +15 °C (Fig. 3) as bright areas, i.e. the higher the free liquid water content, the brighter it appeared. During the discussion of this study, water signal intensity will be considered as free water content. The minimum temperature of the freeze–thaw cycle for MRI measurement was –10 °C. This temperature was sufficient to analyze the changes in free water content, without the death of cells.

Before treatment, water-filled vessels were visible within the xylem (Fig. 3b, 3d) as well as two high-intensity zones (cambium–phloem and pith) and two middle-intensity zones (inner and outer bark; Fig. 3b–d). Longitudinal observation (Fig. 3c) confirmed that the cambium–phloem zone was the most hydrated part.

**Fig. 4.**
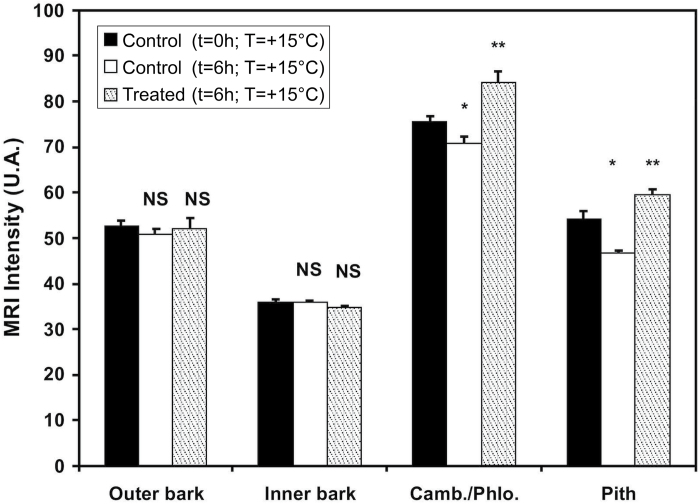
Analysis of MRI images to determine the relative intensity for the four zones indicated in Fig. 3. Means±SE are shown (*n*=12, 4, and 8, respectively) for the control (*t*=0h), the control after 6h at +15 °C, and the treated (*t*=6h) stems after a freeze–thaw treatment (+15 °C/–10 °C/+15 °C). *P≤0.01; **P≤0.05; U.A., arbitrary units; NS, not significant.

After a complete freeze–thaw cycle, the water signal intensity increased significantly in the cambium–phloem and pith areas (Fig. 4). In contrast, the outer and inner bark did not shown any change. In control samples (maintained at a constant temperature of +15 °C for 6h), the water signal intensity decreased in the cambium–phloem and pith areas, while no variation was observed in the outer or inner bark.

### X-ray microtomography observations of frozen and thawed samples

**Fig. 5.**
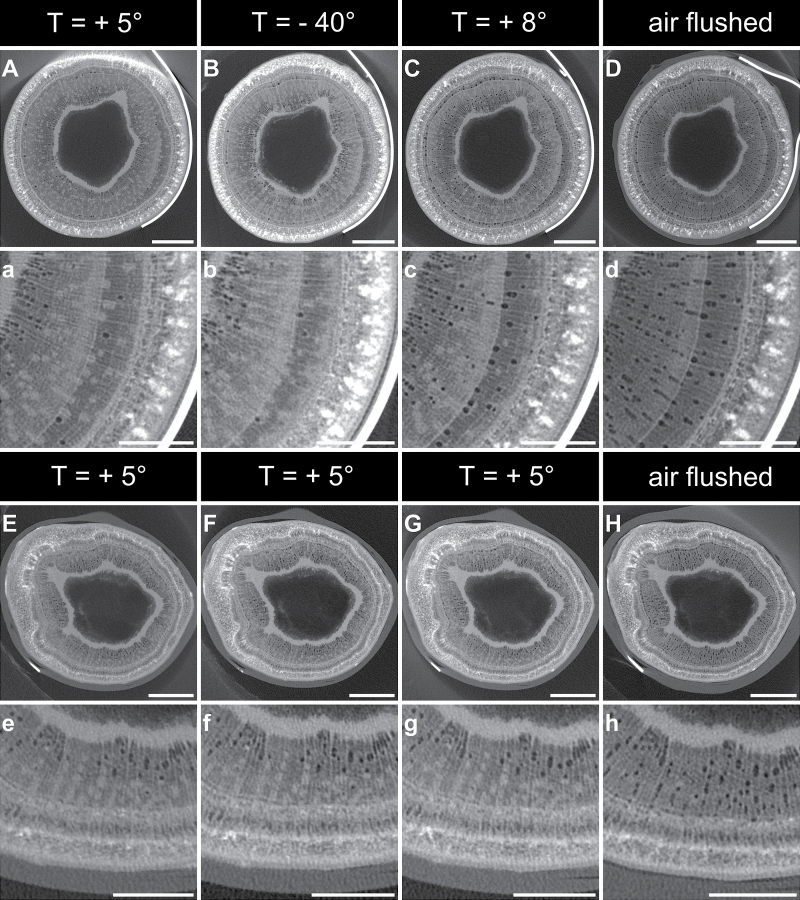
Transverse cross-sections of stem segments observed by X-ray microtomography during a freeze–thaw cycle, showing whole cross-sections (upper rows) and a more detailed view (lower rows). Scans were performed at the temperatures indicated during one freeze–thaw cycle for each sample (*n*=5; A–C, a–c) and control samples (*n*=3; E–G, e–g) were scanned at the same times (*t*=0, 18, and 36h) but were kept at a constant temperature (+5 °C). D and H represent respectively the Scans of air-flushed treated samples and control samples are shown in (D/d) and (H/h), respectively. Bars, 1mm.

**Fig. 6.**
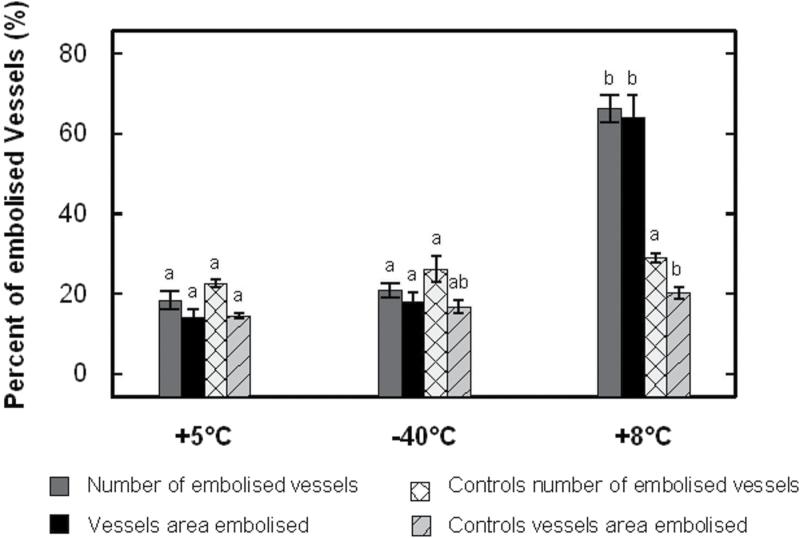
Percentage of embolism at different steps of a freeze–thaw cycle (+5 °C/–40 °C/+8 °C). The number of air-filled vessels was measured by image analysis on X-ray microtomographic scans (*n*=5). For controls (*n*=3), the embolism ratio was determined on scans done at *t*=0, 18 and 36h. Bars with a different letter differed significantly at *P*≤0.05.

The grey levels of three-dimensional images obtained by X-ray microtomography indicate the matter density, i.e. the denser the material, the brighter it appears. Air-filled vessels thus appeared as black spots (Fig. 5). During freezing (from *t*=0 to *t*=18h, corresponding to +5 and –40 °C, respectively), no significant xylem embolism could be detected. X-ray image analysis revealed that, between +5 °C and –40 °C, the number of embolized vessels (18.4±2.2 and 21.0±1.8% at +5 and –40 °C, respectively) and the total area of embolized vessels (14.1±2.2 and 18.0±2.3%, respectively) were not significantly different (Fig. 6). Likewise, no significant difference was observed in control samples, both for number of embolized vessels (22.6±1.1 and 26.2±3.2%, at *t*=0 and 18h corresponding to +5 and –40 °C in freeze–thaw samples, respectively; Fig. 6) and for total area of embolized vessels (14.5±0.7 and 16.8±1.7%; respectively; Fig. 6). Control samples with a constant embolism rate during the whole experiment validated our experiment design with open vessels. Furthermore, the tension of samples was released before experiments by recutting under water several times.

In contrast, embolism in vessels increased during thawing between –40 and +8 °C as shown by X-ray microtomography pictures (Fig. 5, upper row), while no change was observed in control stems at *t*=18 and 36h (corresponding to –40 and +8 °C of treated samples; Fig. 5, lower rows). The number of embolized vessels shifted from 21.0±1.8% at –40 °C (18.0±2.3% in terms of embolized area) to 66.3±3.5% at +8 °C (64.0±5.5% in terms of embolized area; Fig. 6), whereas the number of embolized vessels and the embolized area of the control samples were not significantly different (from 26.2±3.2 and 16.8±1.7% at *t*=18h to 29.0±1.0 and 20.3±1.4% at *t*=36h, respectively; Fig. 6). This observation enabled us to observe for the first time the increase in embolism in one identical stem sample during the thawing process.

### UEs during freezing and thawing

**Fig. 7.**
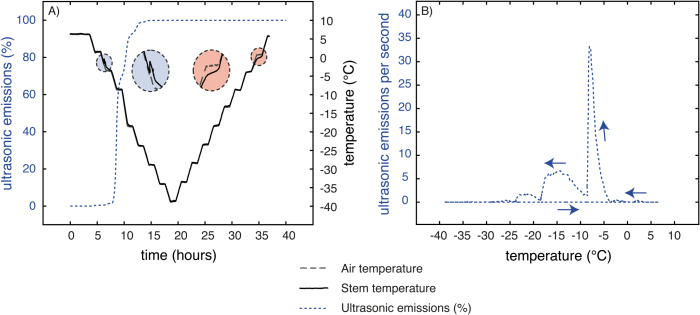
Cumulative UEs during a freeze–thaw cycle. Cumulative UEs were recorded on three samples during a freeze–thaw cycle from 5 down to –40 °C. For details, see Material and methods.

UEs were strictly emitted only during freezing (Fig. 7), starting exactly when the exotherm generated by ice formation was detected. Cumulated UEs increased stepwise following the decreasing temperature steps, and stopped when the temperature reached around –30 °C. Around 80% of UEs were recorded between –5 °C (corresponding to the beginning of the exotherm) and –15 °C, and 100% of UEs were reached at –30 °C (Fig. 7). No UEs were detected during thawing from –40 to +8 °C.

## Discussion

### Freezing

At the onset of ice nucleation, inducing an exotherm at –5 °C (Figs 1 and 7), a drastic radial shrinkage of the stem immediately started (Fig. 1), as also observed previously by Winget and Kozlowski (1964), Zweifel and Häsler (2000), Améglio *et al.* (2001), and Litunen *et al.* (2015). Freezing of acclimated stems induced a combination of living cell shrinkage due to water losses by plasmolysis (Améglio *et al.*, 2001, 2003) or involving putative aquaporine activity (Steppe *et al.*, 2012), and physical shrinkage of cell walls in frozen tissues (Kubler, 1983; Sakai and Larcher, 1987; Zweifel and Häsler, 2000).

Within acclimated stems, as in our experiments, ice nucleation occurs in extracellular spaces, and living cells are dehydrated as protection against frost temperatures (Lu and Rieger, 1993; Rodrigo, 2000; Neuner and Pramsohler, 2006). Water fluxes from living cells to extracellular spaces, resulted in a cell volume drop, which mostly contributed to global stem radial shrinkage (Fig. 1, Table 1). Steppe *et al.* (2012) suggested that, in *J. regia*, the radial hydraulic conductance is variable and is probably mediated by putative aquaporin abundance and/or activity, and that the two possible pathways are the apoplastic and transmembrane routes.

The higher free water content observed in the cambium and pith after a complete freeze–thaw cycle (Fig. 4, Table 1) suggested that ice nucleation took place in these tissues, inducing high Ψ gradients, which drove water from the bark and xylem toward this point and generated strong dehydration during freezing of surrounding tissues (Mazur, 1969; Guy, 1990; Améglio *et al.*, 2001). Améglio *et al.* (2001) found in *J. regia* stems and Ball *et al.* (2004) within non-acclimated leaves that the ice nucleation point is located in the cambium area, which drives water from the bark and xylem to this area.

During freezing, stem shrinkage was caused mainly by bark shrinkage and hardly at all by changes in wood and pith dimension (Fig. 2). The volume variation of ice with temperature (see ‘Thawing’ below) induced a contraction of the bark to a lesser extent. However, bark shrinkage was mainly due to water fluxes leaving the bark from dehydrating living cells (Zweifel and Häsler, 2000; Améglio *et al.*, 2001). The volume changes in wood were small for two reasons: (i) stiffness is much higher in wood than in bark tissues due to lignified cells; and (ii) the low proportion of living cells in wood limits any active movement of water.

No embolism was detected at –40 °C in frozen vessels (Fig. 5), as was also observed with cryoscanning electron microscopy by Utsumi *et al.* (1999) on stems from *Fraxinus mandshurica*, or by Ball *et al.* (2006) on leaves from *Eucalyptus* sp.

UEs were continuously detected during the temperature decrease (Fig. 7, Table 1), as reported in previous studies (Mayr *et al.*, 2007; Mayr and Sperry, 2010; Mayr and Zublasing, 2010). We assume that cavitation events (air bubble formation, a prerequisite for embolism formation) occurred during freezing due to ice-induced high Ψ gradients (Sevanto *et al.*, 2012) and caused UEs, according to Charrier *et al.* (2014a, 2015a) and Ponomarenko *et al.* (2014). Hacker and Neuner (2008) and Pramsohler *et al.* (2012) reported that ice propagation started from the nucleation point and spread along the sample with a higher longitudinal than radial speed, and Charrier *et al.* (2015b) reported that cavitation events occur at the ice front. Although the spatial resolution of our images was too low to detect whether air bubbles were already formed within vessels, such bubbles have been observed previously within conifer xylem (Sucoff, 1969; Robson *et al.*, 1988) and angiosperms leaves (Ball *et al.*, 2006).

Interestingly, 90% of UEs were already emitted and 90% of shrinkage occurred at –15 °C, which is the temperature corresponding to 50% of cell death (LT_50_) within *J. regia* stems in winter (Poirier *et al.*, 2010; Charrier *et al.*, 2011, 2013b). This suggests that at this temperature all extracellular water was frozen, living cells were almost dehydrated, and cavitation occurred within the xylem. At –30 °C, UEs stopped and 98% of shrinkage was observed. During this temperature decrease (from –15 to –30 °C), we assumed that completion of living cell dehydration occurred, some cavitation still took place, and some living cells died, as indicated by the irreversible shrinkage (Améglio *et al.*, 2001, 2003). Finally, at –40 °C, stem shrinkage and UEs tended to stabilize as Ψ gradients were equilibrated (Cochard *et al.*, 2001; Ponomarenko *et al.*, 2014). Theoretically, according to the Clausius–Clapeyron relationship, ice water potential decrease linearly with temperature (–1.16MPa K^−1^ below the freezing point occurring around –5 °C). At –40 °C, ice water potential should reach –40.6MPa. Nevertheless, at this temperature, except for some negligible quantities of water hyperconcentrated within cells, all the water is frozen and the theoretical water potential has a very limited effect and only on the ice contraction, depending on the temperature.

### Thawing

During the temperature increase, stems swelled between –15 and +8 °C (Fig. 1, Table 1). Swelling was stronger as temperatures increased and lower when the temperature remained constant at a plateau, indicating that the diameter variation was directly correlated with temperature changes. Around 80% of the shrinkage and total swelling occurred at the same temperature range, respectively, between the exotherm/endotherm and –15 °C.

The diameter increase from –15 to –5 °C was mainly due to water fluxes toward the cytoplasm of living cells. Indeed, extracellular water with the highest solute concentration, which froze last at around –15 °C, started to thaw first at the same temperature and to refill the most dehydrated living cells with the highest osmotic potential (Fig. 1) (Sevanto *et al.*, 2006). The highest swelling rate was observed between –5 and 0 °C, when most of the ice turned into liquid water, allowing intense water fluxes toward living cells.

From –15 to –5 °C, to a lesser extent, physical dilation of tissues and ice swelling with temperature also increased the stem diameter (Kubler, 1983; Sakai and Larcher, 1987; Zweifel and Häsler, 2000). The linear deformation of ice (in the order of 0.01% K^–1^; Haynes, 2014) would induce a diameter increase of ~10 µm, which is much lower than our observation (diameter increase of ~150 µm).

Similar to the freezing phase, the increase in sample diameter during thawing was mainly due to swelling of the bark (Fig. 2). When the ice turned into water, fluxes moved in the opposite direction from those during freezing, and the water went back into the highly dehydrated living cells of the bark (Uemura *et al.*, 2006). However, after a freeze–thaw cycle down to –40 °C, the bark did not fully recover its initial diameter. This hysteresis suggests that some cells died during the freezing step and could not recover their initial state (Figs 1 and 2; Charrier *et al.*, 2013a, b). Indeed, incomplete recovery of the diameter indicated that temperatures dropped too low or that there was insufficient hardening of samples, leading to damage of living cells (Améglio *et al.*, 2001). Interestingly, in the case of temperature treatment down to –10 °C, the bark fully recovered its free water content after a freeze–thaw cycle (Fig. 4). Indeed, a temperature of –10 °C did not cause cell death; therefore, bark living cells could totally rehydrate themselves to the same state as before the freeze.

After a complete freeze–thaw cycle down to –10 °C, a free water content increase in the cambium–phloem zone as well as in the pith was observed (Fig. 4, Table 1). This may be due to two mechanisms:

(i) Ice formation and/or propagation in the cambium–phloem zone led to partial lysis of cells. Thus, bound water was released and free water appeared on MRI images after a complete freeze–thaw cycle. Nevertheless, this mechanism seems unlikely as living cells should resist lysis down to –10 °C.
(ii) Ice nucleation took place in the cambium and pith regions, and water was then attracted by ice into these tissues during freezing. A complete return of water toward the bark should occur, as cells are still alive after a freeze–thaw cycle down –10 °C. With such temperature treatments, the remaining free water content in the cambium and pith areas should thus come from the wood and particularly from embolism of vessels. We assume that this last mechanism was the main factor inducing the higher free water in the cambium and pith zones after the complete freeze–thaw cycle.

Embolism formation in vessels after a complete freeze–thaw cycle was clearly observed with X-ray microtomography (Fig. 5) as well as with MRI (Supplementary Fig. S1 at *JXB* online). More than 60% of embolism formation was observed after a freeze–thaw cycle down to –40 °C with X-ray microtomography scans (Figs 6 and 7). This consistent with previous work: Charrier *et al.* (2014a) recently reported around 70% embolism after one freeze–thaw cycle down to –40 °C for *J. regia*, 92% was observed in *Quercus robur* (Sperry and Sullivan, 1992), 50–85% in *Fagus sylvatica* (Lemoine *et al.*, 1999), and 40–95% in 10 angiosperm species (Charrier *et al.*, 2014a). In contrast to other studies (Sperry and Sullivan, 1992; Davis *et al.*, 1999; Pittermann and Sperry, 2003; Pittermann and Sperry, 2006), embolism sensitivity did not depend on the vessel diameter in this study, as the integrity of embolism (based on embolized vessel number or embolized area) did not show any significant difference (Fig. 7). Interestingly, embolism occurred after a freeze–thaw cycle down to –10 °C (Supplementary Fig. S1 at *JXB* online, observed by MRI). This is in accordance with Charrier *et al.* (2014a), who reported 40% embolism within *J. regia* stems after one freeze–thaw cycle down to –10 °C.

Many previous studies have reported embolism formation in vessels after a complete freeze–thaw cycle (Améglio *et al.*, 1995; Mayr *et al.*, 2002; Ball *et al.*, 2006; Mayr and Charra-Vaskou, 2007; Charra-Vaskou *et al.*, 2012a). However, development of embolism during thawing was never proved, except by Utsumi *et al.* (1999) on *Fraxinus* stems or Ball *et al.* (2006) on *Eucalyptus* leaves by cryoscanning electron microscopy. In this study, embolism was monitored for the first time on identical samples during the whole freeze-thaw cycle. In accordance with the ‘thaw–expansion hypothesis,’ we clearly demonstrated that embolism occurred during the thawing phase. UEs were emitted only during freezing, indicating that UEs were not directly related to embolism formation.

### Conclusion

The study of events and damages induced by frost is important to understand the physiological processes of trees under current environmental constraints. For the first time, spatial and temporal patterns were monitored during a freeze–thaw cycle on identical tree stems. The use of several non-invasive observation methods gave new insights in the dynamics of processes related to freezing and thawing (Table 1). During freezing, from the time of exotherm formation until –15 °C, water moved mainly from the bark toward the cambium, indicating that ice nucleation occurs in this region. This strong water-attractive point generates high spatial heterogeneity of Ψ gradients within the stem, inducing cavitation of vessels. UEs recorded during freezing are likely to be linked to cavitation events but not to vessel embolism. During thawing, from –15 to +5 °C, water moves back to dehydrated living cells of the bark resulting in stem swelling. Thanks to X-ray microtomography, embolism formation could be visualized during thawing. Embolism development, in contrast to cavitation events, does not induce any UEs. The dissociation of cavitation and embolism events during the freeze–thaw cycle is a great opportunity to analyze, from a physical and physiological point of view, the liquid-to-gas transition, which drives the hydraulic integrity, crucial for the survival of plants.

Use of a microdendrometer allowed us to detect with high precision the exotherm and endotherm temperatures as well as the temperatures when ice propagation ends and thawing starts. Temperature-induced cell damage can also be detected by this non-destructive technique. Microdendrometer and UE techniques, already commonly used for drought stress, could be used as non-destructive methods to monitor the risk of frost damage in the field.

## Supplementary data

Supplementary data are available at *JXB* online.

**Supplementary Fig. S1.** Cross-section MRI images showing emptying of vessels after a freeze–thaw cycle down to –10 °C.

Supplementary Data
