# Supplementary material for: Cavitation and water fluxes driven by ice water potential in Juglans regia during freeze–thaw cycles
Source: J Exp Bot. 2015 Nov 19;67(3):739–50. doi: 10.1093/jxb/erv486 (PMC4737071; doi:10.1093/jxb/erv486)
Supplement: Supplementary Data [file supp_67_3_739__index.html]

Cavitation and water fluxes driven by ice water potential in Juglans regia during freeze–thaw cycles — Cavitation and water fluxes driven by ice water potential in Juglans regia during freeze–thaw cycles — Supplementary Data 

# Cavitation and water fluxes driven by ice water potential in *Juglans regia* during freeze–thaw cycles

## Supplementary Data

Data files

- supplementary\_figure\_S1.pdf - Supplementary Data
