# Supplementary material for: Cavitation and water fluxes driven by ice water potential in Juglans regia during freeze–thaw cycles
Source: J Exp Bot. 2015 Nov 19;67(3):739–50. doi: 10.1093/jxb/erv486 (PMC4737071; doi:10.1093/jxb/erv486)
Supplement: Supplementary Data [file supp_erv486_supplementary_figure_S1.pdf]

Cavitation and water fluxes driven by ice water potential in trees during freeze-thaw cycles.

*Katline Charra-Vaskou, Eric Badel, Guillaume Charrier, Alexandre Ponomarenko, Marc Bonhomme, Loïc Foucat, Stefan Mayr, Thierry Améglio*

## SUPPLEMENTARY DATA

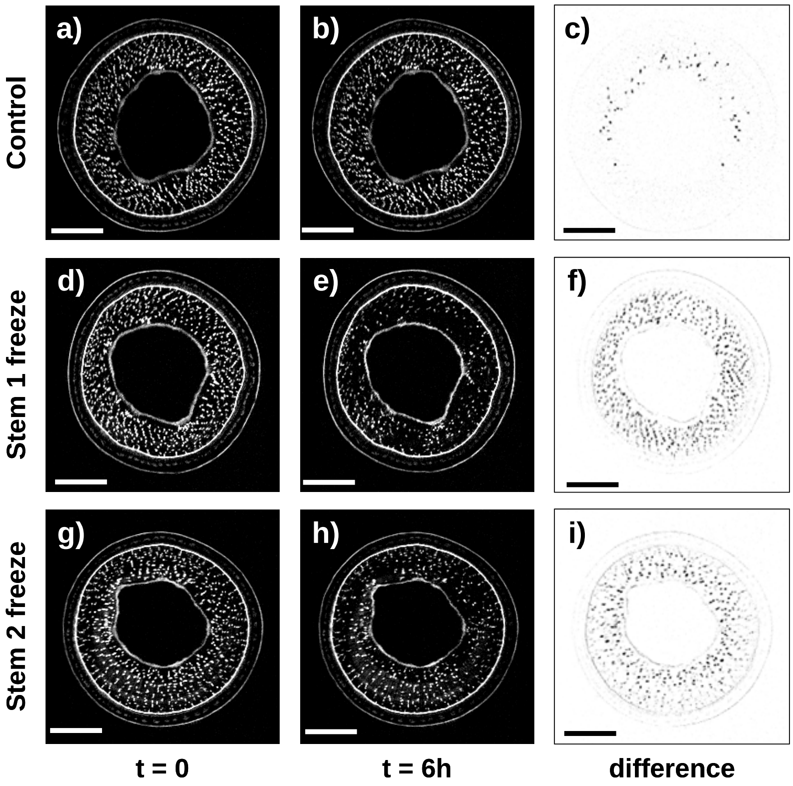


**Supplementary Fig. S1**. Cross section MRI images showing emptying of vessels after control treatment at +15°C and freeze-thaw treatment (+15°C/-10°C/+15°C). Control treatment: -a) at time 0h; -b) 6 hours after; -c) the result of subtraction of image (a) from (b). Dark spot represent vessels that have emptied. Stem 1 freeze: -d) at time 0h; -e) 6 hours after; -f) the result of subtraction of image (d) from (e). Stem 2 freeze -g) at time 0h; -h) 6 hours after; -i) the result of subtraction of image (g) from (h). Scale bars: 2 mm. For control, stem 1 and stem 2, only the first transverse slice was presented.
